# 3D hierarchical porous graphene aerogel with tunable meso-pores on graphene nanosheets for high-performance energy storage

**DOI:** 10.1038/srep14229

**Published:** 2015-09-18

**Authors:** Long Ren, K. N. Hui, K. S. Hui, Yundan Liu, Xiang Qi, Jianxin Zhong, Yi Du, Jianping Yang

**Affiliations:** 1 Hunan Key Laboratory for Micro-Nano Energy Materials and Devices, and School of Physics and Optoelectronics, Xiangtan University, Hunan 411105, People’s Republic of China; 2 Institute of Applied Physics and Materials Engineering, University of Macau, Avenida da Universidade, Taipa, Macau; 3 Department of Mechanical Convergence Engineering, Hanyang University, 17 Haengdang-dong, Seongdong-gu, Seoul 133-791, Republic of Korea; 4 Institute for Superconducting and Electronic Materials, Australian Institute for Innovative Materials, University of Wollongong, Innovation Campus, North Wollongong, New South Wales 2500, Australia; 5 College of Environmental Science and Engineering, State Key Laboratory of Pollution Control and Resources Reuse, Tongji University, Shanghai 200092, China

## Abstract

New and novel 3D hierarchical porous graphene aerogels (HPGA) with uniform and tunable meso-pores (e.g., 21 and 53 nm) on graphene nanosheets (GNS) were prepared by a hydrothermal self-assembly process and an *in-situ* carbothermal reaction. The size and distribution of the meso-pores on the individual GNS were uniform and could be tuned by controlling the sizes of the Co_3_O_4_ NPs used in the hydrothermal reaction. This unique architecture of HPGA prevents the stacking of GNS and promises more electrochemically active sites that enhance the electrochemical storage level significantly. HPGA, as a lithium-ion battery anode, exhibited superior electrochemical performance, including a high reversible specific capacity of 1100 mAh/g at a current density of 0.1 A/g, outstanding cycling stability and excellent rate performance. Even at a large current density of 20 A/g, the reversible capacity was retained at 300 mAh/g, which is larger than that of most porous carbon-based anodes reported, suggesting it to be a promising candidate for energy storage. The proposed 3D HPGA is expected to provide an important platform that can promote the development of 3D topological porous systems in a range of energy storage and generation fields.

Lithium-ion batteries (LIBs) are as a major breakthrough in electrochemical energy conversion and storage devices, and have attracted considerable interest over the past decade^1^,^2^. Despite this, with the development of electronic devices, especially electric vehicles, there has been continuous demand for lighter, longer-lasting and more powerful batteries^3^,^4^. The performance of LIBs is strongly dependent on the characteristics of the electrodes. Therefore, exploring new and novel electrode materials with high capacity, good stability and high rate performance is in urgent demand for the further development of LIBs^5^,^6^,^7^.

Graphite is a commercialized anode because of its good life cycle performance and high columbic efficiency^8^. On the other hand, the specific capacity of graphite is limited to 372 mAh/g by forming an intercalation compound of LiC_6_^9^. To improve the performance of LIBs, anode materials with specific capacities higher than that of graphite are required. A range of carbon nanomaterials, such as carbon nanotubes and graphene, have been explored for this purpose^7^,^9^. For graphene, it possesses larger specific capacity owing to its the huge specific surface area of approximately 2600 m^2^/g and numerous edge sites for hosting lithium^10^,^11^. Moreover, the diffusion distance of lithium ions in nanostructured carbon is short, leading to improved rate performance^7^,^12^. On the other hand, the latest research shows that lithium ions are difficult to stabilize and diffuse in the graphene without defects, and the performance of graphene-based electrodes normally suffers from severe graphene aggregation, which would inevitably hinder inferior ionic accessibility^13^,^14^,^15^. Therefore, a better design of a graphene electrode material is essential to further boosting the performance of graphene in LIBs.

Compared to 2D graphene nanosheets (GNS), micro- or meso-porous GNS has a larger specific surface area and a larger number of additional defects in the basal plane of graphene, which increases the number of electrochemical active reversible storage sites^16^,^17^,^18^,^19^,^20^,^21^. In addition, 2D GNS with well-defined pores (2–300 nm) can accelerate the Li-ion diffusion kinetics further and minimize the Li-ion insertion/extraction distance because of the interconnected channels that connect the interior active sites^22^,^23^,^24^,^25^,^26^. The specific capacity and rate capacity of these 2D porous GNS can reach an extraordinary high level, e.g. 1040 mAh/g at 100 mA/g and 255 mAh/g at 5 A/g^22^. However, the outstanding electrochemical performance of porous GNS still suffers from possible aggregation and the restacking of individual graphene nanosheets^27^,^28^,^29^.

Numerous novel approaches have been explored to inhibit the aggregation and stacking of GNS^30^,^31^,^32^,^33^. This issue may be addressed by the recently developed 3D graphene aerogel (GA), which is assembled by cross-linked individual graphene sheets and exhibiting a continuously interconnected porous network, large surface area, low mass density, and high electrical conductivity^34^,^35^. Based on its structural features, GA is considered to be an ideal prototype for maximization of the accessible surface areas and the development of high-performance electrochemical devices^36^,^37^. On the other hand, 3D GA generally lacks well-defined meso-pores or micro-pores in its structure, which limits substantially the efficiency of mass transport and charge storage for LIBs through the small pores or defects.

Based on the porous GNS and 3D macro-porous GA, hierarchical porous graphene aerogel (HPGA) was constructed here by linking 2D meso-porous GNS to form an interconnected macro-porous GA networks. In previous reports, in addition to 3D macro-porous graphene foams and 2D porous graphene sheets with a pore size in the micrometer range, only a few hierarchical porous graphene nano-materials were produced^29^,^38^,^39^. This is also a challenge to effectively synthesize hierarchical porous graphene nano-materials with both well-defined macro-porous and meso-porous, particularly with a controlled pore size, which may open new applications in various energy fields. This paper reports a simple and efficient approach to fabricate the free-standing HPGA with uniform and tunable meso-pores (e.g., 21 and 53 nm) on GNS. First, 20 nm and 50 nm Co_3_O_4_ NPs were loaded uniformly on 3D GA via a hydrothermal process. The meso-pores on GNS were then produced by the decomposition of the carbon source via an *in-situ* carbothermal reaction. By combining the synergistic effects of the macro-porosity of the GA network architecture and the meso-porosity of GNS, the as-prepared HPGA samples possessed a high density of ion diffusion channels that facilitate charge transport and storage at high rates, which results in high Li ions storage capacities for more than 100 cycles of continuous charge/discharge (1100 mAh/g at current density of 0.1 A/g). To the best of the authors’ knowledge, this is the first demonstration of 3D GA with a hierarchical porous architecture in lithium-ion batteries. HPGA may be an effective electrode material for other high-performance energy storage and generation devices.

## Results and Discussions

### Material preparation and characterization

**Figure 1:**
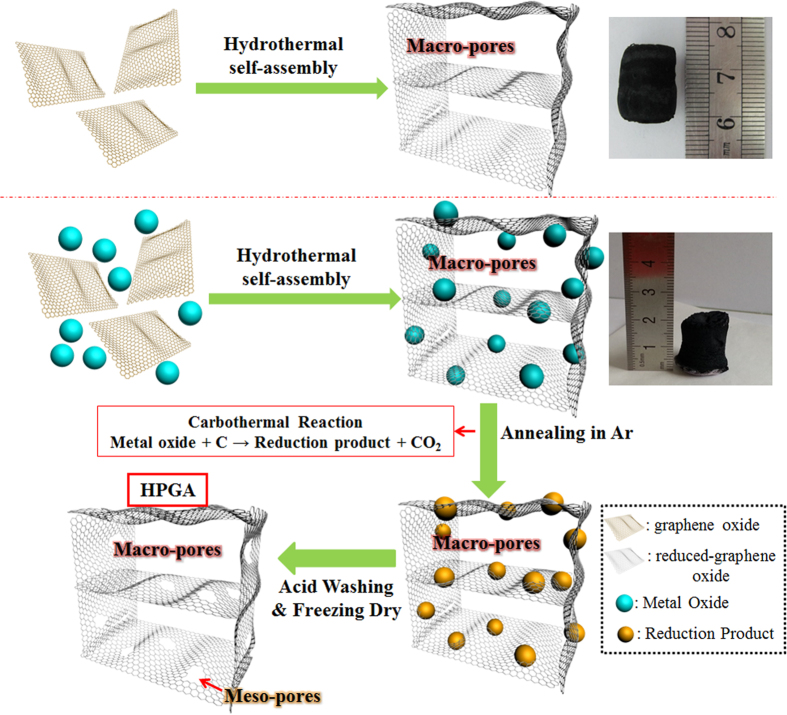
Schematic diagram of the morphological formation of HPGA.

Figure 1 presents a schematic diagram of the methodology for preparing 3D GA and HPGA. A hydrothermal self-assembly process was first carried out to load metal oxides Co_3_O_4_ NPs (20 nm or 50 nm) on graphene oxide (GO), which was then reduced to form 3D GA. During the reduction of a GO solution in the hydrothermal environment, hydrophilic GO sheets were transformed into hydrophobic graphene sheets. The oxygenated functionalities decreased significantly and the π-conjugation was largely restored which results in increased incompatibility with polar solvents and π−π stacking interaction^33^,^34^. At the same time, with the help of electrostatic attraction and physical entrapment, metal oxide nanoparticles dispersed homogeneously in the aqueous suspension could be embedded into and captured by the graphene network to form a 3D graphene/nanoparticles gel. After freeze-drying, the Co_3_O_4_/GA samples with high interconnected macro-porosity and uniform deposition of Co_3_O_4_ NPs were obtained. Subsequently, the GA samples were annealed at 900 °C under a protective atmosphere (Ar gas). During this process, due to a carbothermal reaction between Co_3_O_4_ NPs and the carbon materials, the Co_3_O_4_ NPs were reduced, and the GNS, as the carbon source, was decomposed and etched *in situ* to form meso-pores on the plane. After washing with diluted HCl acid (10%) three times to remove the reduction products of Co_3_O_4_, followed by freeze-drying for 12 hours, HPGA with well-defined meso-pores on GNS and macro-pores was prepared. The sizes of these meso-pores could be tuned by controlling the size of the Co_3_O_4_ NPs.

**Figure 2:**
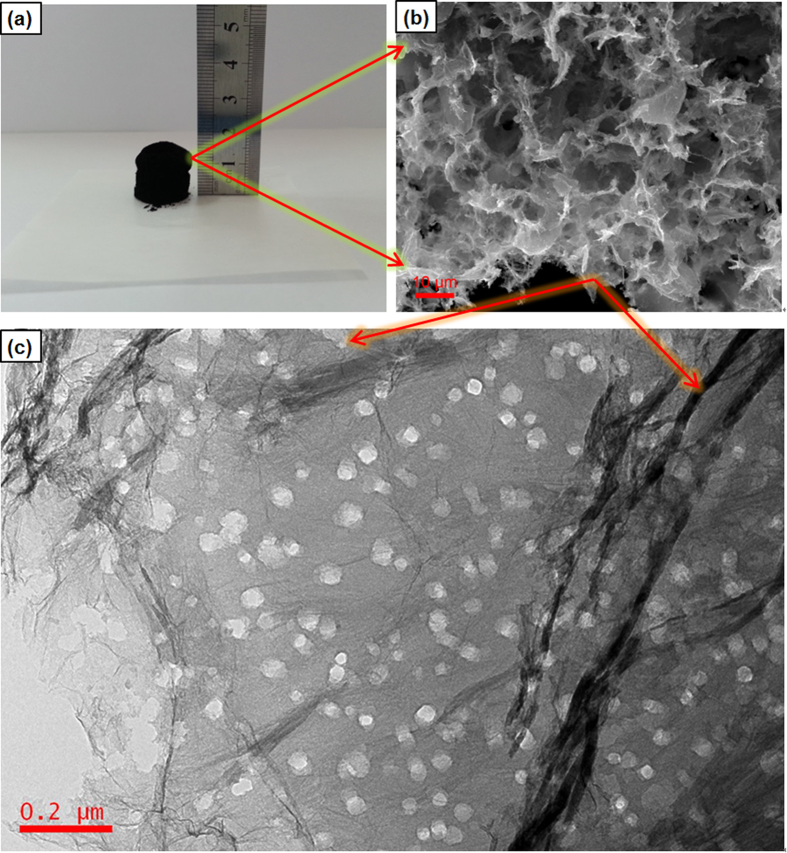
(**a**) Digital images of the as-prepared HPGA-50, (**b**) SEM image of HPGA-50, (**c**) TEM image of HPGA-50.

As shown in Fig. 2a, a free-standing cylindrical shape 3D HPGA-50 with a diameter and height of 10 mm and 20 mm, respectively, was obtained (50 refers to 50 nm Co_3_O_4_ NPs used in the process). From the SEM image of HPGA-50 (Fig. 2b), a macro-porous structure consisting of interconnected networks of randomly oriented sheet-like structures was clearly observed. These sheets were rather thin and wrinkled, indicating the efficient self-assembly of GNS. Digital photos and SEM images confirm that the profile and internal structure of the as-prepared 3D HPGA-50 architecture was similar to that of its precursors (see SEM images of the precursors of HPGA-50, Figure S1), without significant volume loss after the carbothermal reaction and acid washing process. The structural features of the prepared HPGA-50 were also similar to those reported elsewhere^34^,^40^; however, the sub-microstructure of the HPGA-50 was quite different and interesting. In particular, TEM (Fig. 2c) revealed densely distributed pores emerged in the local microscopic region of 3D HPGA network. In contrast, as shown in Figure S2, there was no pore on the GNS in the GA sample which also has a similar macro-porous structure. Those pores on the 2D GNS which constructed HPGA were well-defined and distributed uniformly. The mean size of the pores in the GNS was 53 nm (the statistical results can be found in the supplementary information, Figure S3b), which is slightly larger than the size of the Co_3_O_4_ NPs (50 nm) used in the preparation.

The formation mechanism of the meso-pores on GNS is proposed as an *in situ* carbothermal reaction between Co_3_O_4_ NPs and carbon. XRD (Figure S4) showed that the Co_3_O_4_/GA prepared from the initial hydrothermal process was converted to Co/CoO/GA after the annealing procedure. A carbothermal reduction of Co_3_O_4_ occurred in the annealing procedure, which consumed the carbon of GA, as a reducing agent, to produce carbon monoxide or carbon dioxide, and the Co_3_O_4_ was reduced to the metal or lower valence state metal oxides^41^. The carbothermal reduction reaction of Co_3_O_4_ can be expressed as Eq. (1):


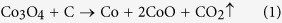


**Figure 3:**
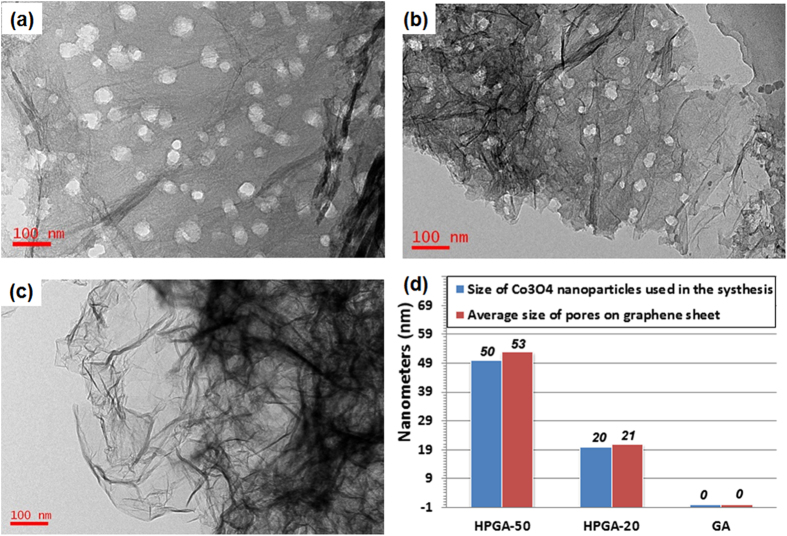
TEM images of HPGA-50 (a), HPGA-20 (b), GA (c) and their pore size measurements (d).

**Figure 4:**
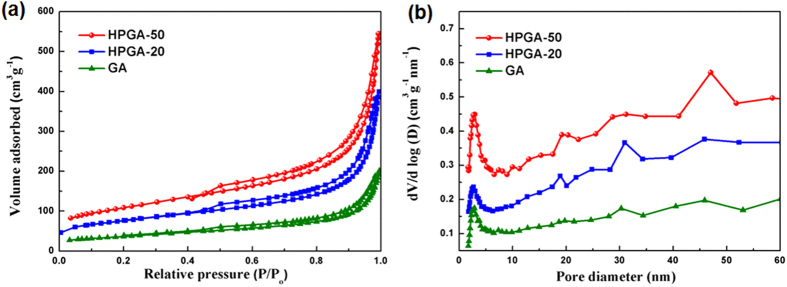
(**a**) Nitrogen adsorption and desorption isotherms and (**b**) BJH pore size distribution of HPGA-50, HPGA-20 and GA.

The carbon atoms on the GNS were sacrificed in the reaction (Eq. 1). The meso-pores on the graphene plane were produced by removing the Co and CoO from the GA by dilute HCl acid. The hypothesis was that the size of the meso-pores on the graphene plane can be controlled by changing the size of the Co_3_O_4_ NPs in the preparation method. Another HPGA-20 sample was prepared to show that the proposed method is effective in producing a HPGA architecture with controllable pore sizes in the GNS. As shown in Figures S2c and S2d, well-defined meso-pores with a mean diameter of 21 nm were distributed uniformly over the GNS. Figure 3a–c shows TEM images of HPGA-50, HPGA-20 and GA. Figure 3d presents the measured mean size (see Figure S3) of the pores on the GNSs. Various HPGA samples with different meso-pore sizes on the GNS can be prepared using different sizes of Co_3_O_4_ NPs or other metal oxides, and the distribution density of the meso-pores on the graphene can be also changed by different Co_3_O_4_/graphene ratio used in the experiment (see the Figure S5). To further characterize the porous structure of as-prepared samples, the results about N_2_ absorption/desorption analysis of the novel HPGA-50, HPGA-20 and conventional graphene aerogel (GA) were shown in Fig. 4. Based on Brunauer-Emmett-Teller (BET) method, the specific surface areas (SSA) of HPGA-50, HPGA-20 and GA are 383.7 m^2^/g, 266.4 m^2^/g and 130.1 m^2^/g, respectively. The hierarchical porous graphene aerogel with tunable meso-pores (HPGA-50 and HPGA-20) have huger SSA than the conventional graphene aerogel without meso-pores. The HPGA-50 with bigger pore contains largest SSA. The BET results are consistent with the internal nanostructure which mentioned in this paper. Besides the specific surface areas analysis, the pore size distributions of HPGA-50, HPGA-20 and GA were also derived from the adsorption branches of isotherms by using the Barrett-Joyner-Halenda (BJH) model. All the three porous graphene aerogel showed a prominent pore size distribution in the range of 2–3 nm, which could be ascribed to the nanopores in the basal plane of GNS. Particularly, HPGA-50 and HPGA-20 showed another prominent pore size distribution around 48 nm and 30 nm, respectively, which agreed with the statistical pore-size results from the TEM of HPGA-50 and HPGA-20.

**Figure 5:**
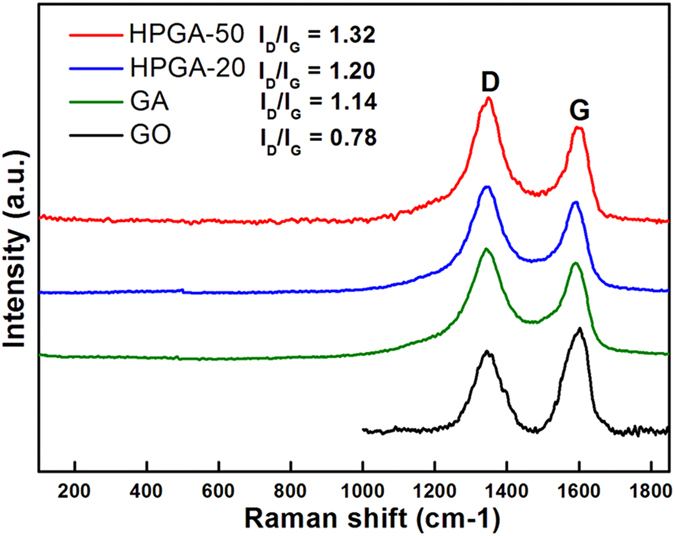
Raman patterns of HPGA-50, HPGA-20, GA and GO.

The graphene nature of the prepared HPGA samples was confirmed further by Raman spectroscopy (Fig. 5). The Raman band at approximately 1590 cm^−1^ corresponds to the first order scattering of the E2g phonon of sp2 C atoms (the G band), and the peak around 1350 cm^−1^ was derived from the breathing mode of the aromatic rings (the D band). The D/G intensity ratios of the HPGA-50, HPGA-20 and GA (without pores on the GNS) were larger than that of GO (1.32 for HPGA-50, 1.20 for HPGA-50, 1.14 for GA and 0.78 for GO), which is consistent with the typical Raman spectra of GO and GNS^42^. The more intense D band, as shown in the HPGA-50 and HPGA-20 spectrum, indicates the partial lattice defects of the meso-porous GNS of the HPGA samples compared to the graphene layers without porosity^22^,^43^. In contrast to HPGA-20, HPGA-50 showed a higher D/G intensity ratio, suggesting more defects in the sample, which might be due to the larger pores in the HPGA-50.

### Battery testing

**Figure 6:**
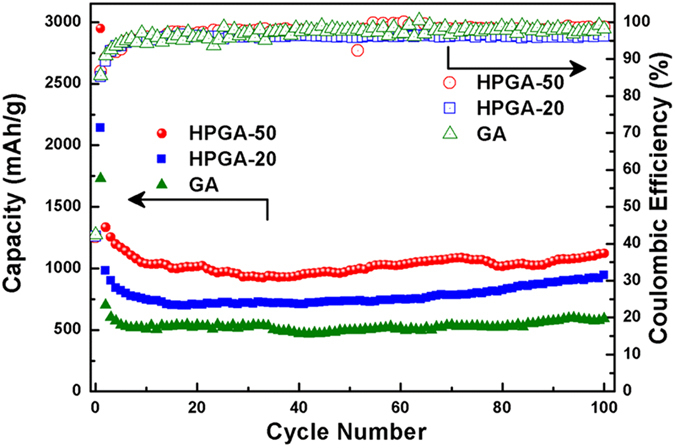
Cycling performance and coulombic efficiencies of HPGA-50 and HPGA-20 anodes compared to GA at 0.1 A/g with a voltage window of 0.1–3.0 V.

Owing to the hierarchical porous GA framework, and abundant meso-pores on GNS for ion diffusion and adsorption, the HPGA samples can serve as a promising substitute for the graphite anode in LIB applications. The HPGA samples were fabricated as a LIB anode based on a standard fabrication procedure, and their electrochemical storage capacities were evaluated based on the half-cell configuration^44^. A normal GA without meso-pores was also fabricated as LIB anodes for comparison. Figure 6 shows the cycling performance and coulombic efficiencies of the three electrodes at a current density of 100 mA/g. All the three samples (HPGA-50, HPGA-20, GA) exhibit an initial Coulombic efficiency of approximately 42% and a subsequent Coulombic efficiency of over 96%. These results are comparable to the high Coulombic efficiency of commercial graphite and the former reported graphene anodes^22^,^45^. For the HPGA samples, a high reversible discharge capacity of 1100 mAh/g (HPGA-50) and 700 mAh/g (HPGA-20) was retained after 100 charge–discharge cycles, which are superior to that of the GA sample and the reported graphite anode^9^. Compared to the GA anode, the HPGA samples (HPGA-50 and HPGA-20) exhibited a substantially higher lithium ion storage capacity and excellent cycling stability. This excellent electrochemical property was attributed to the hierarchical structure of HPGA with meso-pores on GNS. Lithium ions can be adsorbed electrochemically on and intercalated into both sides of the free-standing thin graphene networks, as well as the mesopores on the graphene planes, thereby providing a higher reversible Li^+^ storage capacity than the GA with only macro-porous frameworks. Although the meso-pores on the GNS of the two HPGA samples provided efficient transport pathways for ion diffusion toward the deep portions of the stacked graphene layers, the results indicated that the HPGA-50 anode with larger meso-pores had a higher reversible capacity and better cycling stability (Fig. 6).

**Figure 7:**
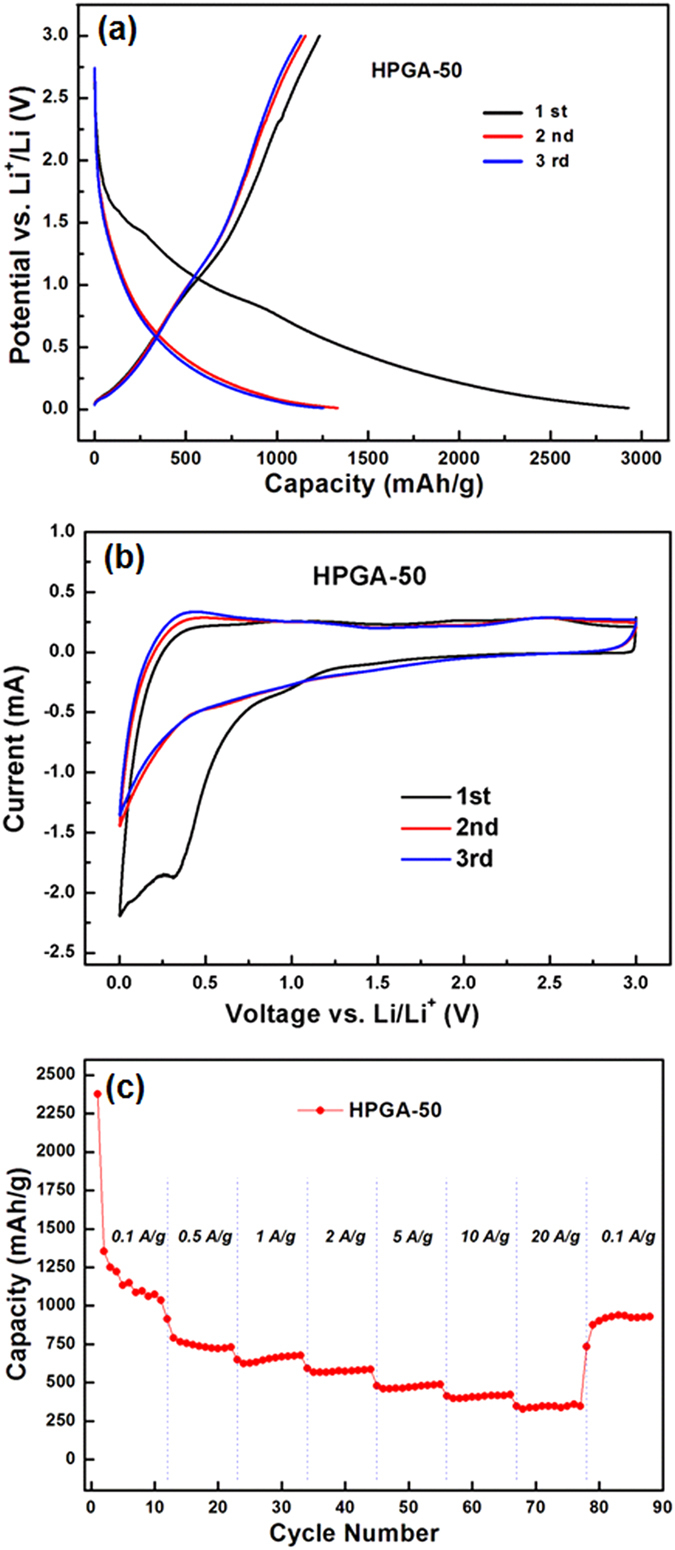
(**a**) Discharge/charge voltage curves for the first three cycles of HPGA anode at 0.1 A/g with a voltage window of 0.1–3.0 V. (**b**) First three cyclic voltammograms of HPGA anode at a scan rate of 1 mV/s between 0.1–3.0 V. (**c**) Rate capability of the HPGA-50 anode.

For the HPGA-50 anode, at a current density of 100 mA/g, an ultrahigh capacity of approximately 2900 mAh/g was recorded in the initial discharge, which decreased to 1400 mAh/g in the second cycle (Fig. 7a). The capacity loss after the first cycle, which has been observed for many porous carbon or graphene-based materials, is due mainly to the decomposition of the electrolytes and the formation of a solid−electrolyte interface (SEI) on the surface of the electrode materials^12^. For the HPGA-50 anode, the ultrathin layer of GNS and the hierarchical porous structure greatly improved the efficient contact between the electrolyte and the electrode, resulting in enhanced deposition of the SEI layer on the electrode surface.

According to the galvanostatic discharge/charge curves (Figure S6), the initial capacity of the HPGA-20 anode was slightly lower than that of HPGA-50, which may be due to the fewer activated sites with smaller meso-pores on GNS. Moreover, the irreversible capacity loss of HPGA-50 (1600 mAh/g) in the first cycle was larger than that of HPGA-20 (1200 mAh/g), which may be due to the larger SEI layer formed by the larger holes on the sample. On the other hand, the initial irreversible capacity loss did not have a significant influence on the higher reversible capacity of the HPGA-50 anode in the cycle test. The meso-pores on GNS are expected to create numerous new Li^+^ storage sites, but these meso-pores may be blocked by the SEI layer. These results suggest that the larger meso-pores in the HPGA-50 anode probably remained unblocked, leading to abundant activity on the Li^+^ storage site. In contrast, the smaller meso-pores of the HPGA-20 anode were likely to be blocked by the SEI layer, leading to fewer storage sites and lower reversible capacity.

The CV plot reveals distinct reduction peaks related to the irreversible capacity loss in the first cycle (Fig. 7b), which is in good agreement with the first galvanostatic discharge/charge curve (Fig. 7a). The results suggest that structural defects in the HPGA-50 anode may also contribute to the irreversible capacity, due to the pseudo-capacitive effect. But no obvious changes were observed from the second to the fifth cycle of the CV plots, indicating good cycling stability. Figure 7c shows the cycling performance of the HPGA-50 anode, which was evaluated by galvanostatic cycles at different charge/discharge rates. At a current density of 0.1 A/g, the reversible capacity of the HPGA-50 anode was approximately 1050 mAh/g, which is 3 times higher than the value of graphite (372 mAh/g^9^). After increasing the discharge/charge current density to 0.5, 2 and 10 A/g, however, the reversible capacity was stabilized at 750, 500 and 400 mAh/g, respectively. At an ultrahigh current density of 20 A/g (~50C), a large reversible capacity of 300 mAh/g with excellent cycling stability was obtained. The ultrahigh rate performance should be due to the unique ultrathin GNS layer structure with abundant and uniform meso-pores and a 3D macro-porous graphene network, which reduces the lithium ion diffusion length and enhances lithium ion diffusion. Table 1 compares the capacity of the different graphene-based anodes produced, which confirmed that HPGA-50 has promising high rate performance.

**Figure 8:**
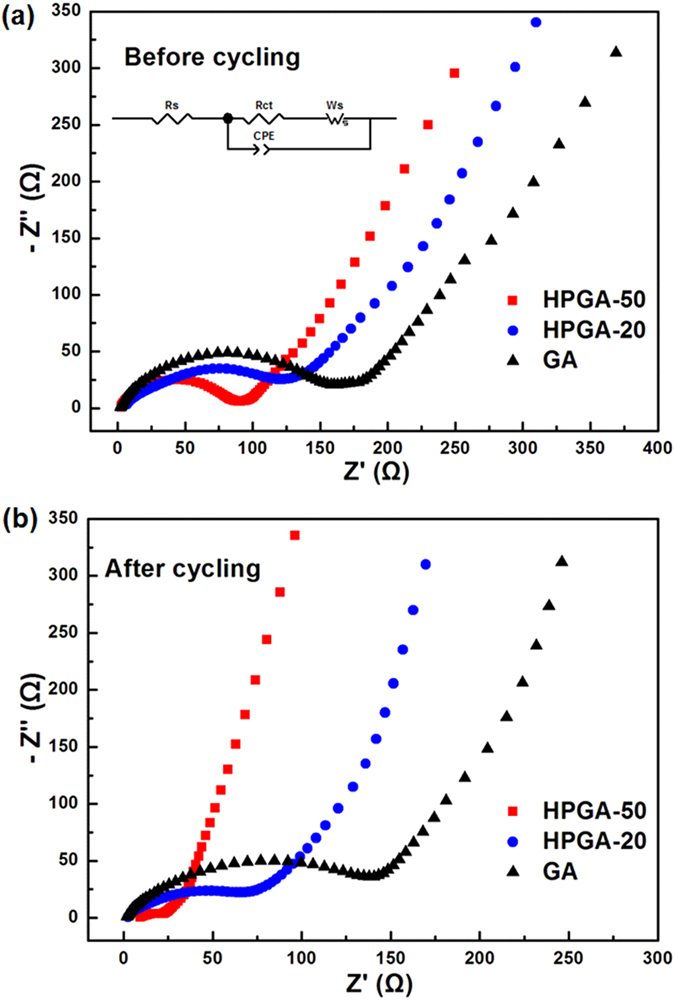
Typical electrochemical impedance spectra, presented as Nyquist plots for HPGA-50, HPGA-20 and GA anode before (**a**) and after (**b**) 100 discharge/charge cycling.

To further examine the mechanisms contributing to the high capability and superior cycling stability of the HPGA samples, electrochemical impedance spectroscopy (EIS) was carried out on the HPGA-50, HPGA-20 and GA anodes before and after 100 charge/discharge cycles at 100 mA/g. The Nyquist plots of the samples (Fig. 8) showed a broad depressed semicircle at the high-frequency region corresponding to the interfacial resistance and charge-transfer kinetic-controlled region^46^,^47^. The straight line at the low-frequency region is associated with the Warburg region and can be attributed to the diffusion of lithium ions in the GNS^45^. All the data was quantified by fitting to a typical equivalent circuit for battery electrodes (inset in Fig. 8). Table 2 lists the charge-transfer resistance, constant phase element and Warburg coefficient. As shown in Fig. 8 and Table 2, the semicircles at high-frequency before cycling gradually decreased in size from the GA, HPGA-20 to HPGA-50, showing a substantial reduction in charge-transfer resistance with increasing pore size on GNS. After cycling, despite the reduced charge-transfer resistance of each sample due to the formation of a SEI layer, the charge-transfer resistance also decreased with increasing pore size of GNS in the samples. The charge-transfer resistance of the HPGA-50 anode was significantly lower after cycling, indicating that more SEI layers may be generated on the HPGA-50 caused by the larger pores on this sample. The smaller decrease in charge-transfer resistance of GA before and after cycling might be caused by the relatively few SEI layers formed on the GA sample. In the equivalent circuit, the constant phase element (CPE) represents the electric double-layer capacitance of nonhomogeneous systems^37^,^48^. In detail, the values of CPE-T are related to the capacitance of the HPGA and GA anodes in this electrochemical system. As shown in Table 2, the CPE-T values of two HPGA samples are larger than the GA sample. The HPGA-50 possessed the highest capacitance among the electrodes before and after cycling. The capacitance is related to the roughness of the electrode surface and porosity, as well as to the adsorption/desorption processes between the electrode and electrolyte^48^. This suggests that higher capacitance can be obtained with increasing porosity in the HPGA sample. In addition, the decrease in the capacitance of all electrodes after cycling could be due to the formation of the SEI layer. The Warburg coefficients of the samples before and after cycling also decreased with increasing pore size on GNS, suggesting lower ion diffusion resistance for the HPGA samples, particularly the HPGA-50 anode^45^. The EIS measurements confirmed the remarkably improved charge-transfer and Li-ion diffusion kinetics in the HPGA-50 anode, suggesting that the HPGA-50 electrode possesses high electrical conductivity and undergoes a rapid charge transfer reaction with fast lithium ion diffusion.

## Conclusion

Novel free-standing 3D HPGA with uniform and tunable meso-pores on GNS were prepared via a hydrothermal self-assembly process with a subsequent *in situ* carbothermal reaction. The proposed method is simple and environment friendly. The meso-pores on the GNS of HPGA can be controlled using Co_3_O_4_ NPs with a suitable size. This unique 3D macro-porous architecture of GA with 2D meso-porous GNS provides the networks for adsorption, thin graphitic layers for Li ion intercalation, and abundant meso-pores as cavities to maximize the adsorption of Li ions, leading to remarkable electrochemical properties. The HPGA-50 anode exhibited superior electrochemical performance including a high reversible specific capacity of 1100 mAh/g at a current density of 0.1 A/g, outstanding cycling stability and excellent rate performance. Even at a large current density of 20 A/g, the reversible capacity was approximately 300 mAh/g, which is larger than many porous carbon-based anodes reported elsewhere. The proposed HPGA samples may lead to further developments of new device concepts and architectures, thus allowing more opportunities to realize novel electrochemical storage with higher power and energy densities.

## Methods

### Materials

Graphite flakes were purchased from Sinopharm Chemical Reagent Co. Ltd. The 20 nm Co_3_O_4_ NPs were acquired from Alfa Aesar (CAS: 1308-06-1) and the 50 nm Co_3_O_4_ NPs were obtained from Nanjing Emperor. All the other reagents were of analytical purity and used as received from Shanghai Chemical Company.

### Preparation of HPGA

First, the Co_3_O_4_/graphene hydrogel (GH) was prepared via a hydrothermal process. Graphene oxide (GO) was prepared by the oxidation of graphite flakes according to the modified Hummers’ method. In detail, 30 ml of a GO (brown colloidal) dispersion (3 mg/ml) was prepared by an ultrasonic treatment of GO in deionized water for approximately 1 h at room temperature. Subsequently, 0.5 mmol (0.12 g) of Co_3_O_4_ NPs (mean diameter: 20 nm or 50 nm) was added to the obtained GO colloidal dispersion and stirred for a further 2 h to produce a homogeneous suspension. The suspension was then placed in a 50 mL Teflon-sealed autoclave and maintained at 180 °C for 12 h. Finally, after cooling to room temperature, a columnar 3D GH loaded with Co_3_O_4_ NPs was obtained. The free-standing Co_3_O_4_/GH was then removed and freeze-dried for 12 h to yield the Co_3_O_4_/graphene aerogel (GA).

Second, the as-prepared Co_3_O_4_/GA was annealed at 900 °C (rate of temperature rise: 10 °C/min) in an Ar atmosphere for 2 h. The carbothermal reduction reaction of Co_3_O_4_ was carried out using this process. The GA loaded with the reduced-Co/CoO NPs was then obtained. The reduced-Co/CoO NPs were removed by a dilute hydrochloric acid (10%) treatment. Finally, the final HPGA sample was obtained after washing the sample several times with distilled water followed by freeze-drying. HPGA-50 and HPGA-20 were prepared using 50 nm and 20 nm Co_3_O_4_ NPs, respectively, in the hydrothermal process.

### Characterization

The as-prepared samples were characterized by transmission electron microscopy (TEM, JEM 2100), scanning electron microscopy (SEM, JEOL, JSM-6360) equipped with energy dispersive spectroscopy (EDS) components, Raman spectroscopy (Renishaw In-Via, 532 nm), and X-ray diffraction (XRD) using Cu Kα radiation. The porosity was measured by nitrogen sorption isotherms at 77 K with a Micromeritics Tristar 3020 analyzer (USA). Before measurements, the samples were degassed in vacuum at 180 °C for at least 6 h. The Brunauer-Emmett-Teller (BET) method was utilized to calculate the specific surface areas, using adsorption data in a relative pressure (P/P_0_) range from 0.04 to 0.2. The pore volume and pore size distributions were derived from the adsorption branches of isotherms by using the Barrett-Joyner-Halenda (BJH) model. The total pore volume, V_t_, was estimated from the amount adsorbed at a relative pressure P/P_0_ of 0.995.

### Electrochemical Measurements

The electrochemical characterization was carried out in a CR 2023-type coin cell. The working electrodes were prepared using a slurry coating procedure. In detail, a slurry, which consisted of an active material, acetylene black and polyvinylidene fluoride (PVDF) at a weight ratio of 8:1:1 mixed with a suitable amount of N-methyl pyrrolidinone (NMP), was spread uniformly over a copper foil current collector. For a typical preparation of the anode in CR 2023-type coin cell, the mass loading of active materials is around 0.3 mg/cm^2^, which is comparable to the amount previously reported for other porous anode materials^49^. The electrode was dried under vacuum at 120 °C for 12 h. The half-cells were assembled in an argon-filled glovebox with lithium foil as both the counter and reference electrodes and a polypropylene (PP) film as the separator. The electrolyte was a 1 M LiPF6 solution in ethylene carbonate and diethyl carbonate (w/w = 1:1). Cyclic voltammetry was carried out between 3.0 and 0.1 V at a scan rate of 1 mV/s on an electrochemical workstation (CHI 660D). The electrochemical impedance measurements were also carried out using the CHI 660D electrochemical workstation system by applying 100 KHz to the 0.01 Hz frequency ranges with AC oscillation amplitude of 5 mV. The galvanostatic charge–discharge experiments were performed on a Newware battery testing system (Shenzhen Newware Technology Co., Ltd.) at different current rates with a voltage window of 0.1–3 V.

## Additional Information

**How to cite this article**: Ren, L. *et al.* 3D hierarchical porous graphene aerogel with tunable meso-pores on graphene nanosheets for high-performance energy storage. *Sci. Rep.* **5**, 14229; doi: 10.1038/srep14229 (2015).

## Supplementary Material

Supplementary Information

## Figures and Tables

**Table 1:** Performance comparison of the graphene-based anodes.

**Anode material**	**Reversible capacity**	**Current density**	**work**
Graphene sheet	730/560 mAh/g	74/744 mA/g	12
Photoreduced graphene sheets	156 mAh/g	40C	45
Porous graphene networks	926/240 mAh/g	1C/20C	24
Mesoporous graphene nanosheets	833/255 mAh/g	100/5000 mA/g	22
Doped hierarchically porous graphene electrode	510/380 mAh/g	1000/5000 mA/g	15
HPGA-50	1100/300 mAh/g	100/20000 mA/g	This work

**Table 2:** Calculated charge-transfer resistance, constant phase element and Warburg coefficient (at f = 0.16 Hz) of HPGA-50, HPGA-20 and GA electrodes.

	**Before cycling**			**After cycling**		
**Sample**	**Charge-transfer resistance (ohm)**	**Constant phase element (T, farad)**	**Warburg coefficient (ohm·s^−1/2^)**	**Charge-transfer resistance (ohm)**	**Constant phase element (T, farad)**	**Warburg coefficient (ohm·s^−1/2^)**
**HPGA-50**	85	4.5 × **10**^**−5**^	4.9 × **10**^**−2**^	15	8.3 × **10**^**−4**^	5.4 × **10**^**−2**^
**HPGA-20**	137	6.1 × **10**^**−5**^	2.1 × **10**^**−3**^	79	8.9 × **10**^**−5**^	2.3 × **10**^**−3**^
**GA**	160	3.9 × **10**^**−5**^	4.2 × **10**^**−3**^	148	3.2 × **10**^**−5**^	7.4 × **10**^**−3**^
