# Corrigendum: 3D hierarchical porous graphene aerogel with tunable meso-pores on graphene nanosheets for high-performance energy storage

**DOI:** 10.1038/srep17243

**Published:** 2016-01-07

**Authors:** Long Ren, K. N. Hui, K. S. Hui, Yundan Liu, Xiang Qi, Jianxin Zhong, Yi Du, Jianping Yang

Scientific Reports 5: Article number: 1422910.1038/srep14229; published online: 09182015; updated: 01072016.

This Article contains typographical errors in Table 2.

Under the column ‘Before Cycling’, the Warburg coefficient (ohm·s^−1/2^) for samples ‘HPGA-50 (4.9 × 10^2^)’, ‘HPGA-20 (2.1 × 10^3^)’ and ‘GA (4.2 × 10^3^)’ were incorrectly given as ‘HPGA-50 (4.9 × 10^−2^)’, ‘HPGA-20 (2.1 × 10^−3^)’ and ‘GA (4.2 × 10^−3^)’ respectively.

In addition, under the column ‘After Cycling’, the Warburg coefficient (ohm·s^−1/2^) for samples ‘HPGA-50 (5.4×10^2^)’, ‘HPGA-20 (2.3×10^3^)’ and ‘GA (7.4 × 10^3^)’ were incorrectly given as ‘HPGA-50 (5.4×10^−2^)‘, ‘HPGA-20 (2.3×10^−3^)’ and ‘GA (7.4×10^−3^)’ respectively.

