# Supplementary material for: 3D hierarchical porous graphene aerogel with tunable meso-pores on graphene nanosheets for high-performance energy storage
Source: Sci Rep. 2015 Sep 18;5:14229. doi: 10.1038/srep14229 (PMC4585608; doi:10.1038/srep14229)
Supplement: Supplementary Information [file srep14229-s1.doc]

# Supplementary Information

# for

**3D hierarchical porous graphene aerogel with tunable meso-pores on graphene nanosheets for high-performance energy storage**

Long Ren ^1,4*^, K.N. Hui ^2*^, K.S. Hui ^3*^, Yundan Liu ^1^, Xiang Qi ^1^, Jianxin Zhong ^1^, Yi Du ^4^, Jianping Yang ^4,5^

^1^ Hunan Key Laboratory for Micro-Nano Energy Materials and Devices, and School of Physics and Optoelectronics, Xiangtan University, Hunan 411105, People's Republic of China

^2^ Institute of Applied Physics and Materials Engineering, University of Macau, Avenida da Universidade, Taipa, Macau.

^3^ Department of Mechanical Convergence Engineering, Hanyang University, 17 Haengdang-dong, Seongdong-gu, Seoul 133-791, Republic of Korea

^4^ Institute for Superconducting and Electronic Materials, Australian Institute for Innovative Materials, University of Wollongong, Innovation Campus, North Wollongong, New South Wales 2500, Australia

^5^ College of Environmental Science and Engineering, State Key Laboratory of Pollution Control and Resources Reuse, Tongji University, Shanghai 200092, China

^*^ Correspondence and requests for materials should be addressed to Long Ren (email: longren@xtu.edu.cn) or K.N. Hui (email: bizhui@umac.mo) or K.S. Hui (email: kshui@hanyang.ac.kr).

**Figure captions**

Figure S1. Digital Photos and SEM images of the precursors of HPGA-50 after the hydrothermal process (a, b), after the annealing process (c, d), and acid washing process (e, f).

Figure S2. Digital Photos and SEM images of GA without meso-pore graphene sheets after the hydrothermal assembly process of graphene oxide.

Figure S3. TEM images and statistical pore size results of HPGA-50 (a, b) and HPGA-20 (c, d).

Figure S4. XRD pattern of the as-prepared sample in different stages.

Figure S5. TEM images of HPGA samples prepared by 50 nm Co_3_O_4_ nanoparticles but different Co_3_O_4_/GO ratio (weight ratio: (a) 0.06g/0.09g, (b) 0.12g/0.09g, (c) 0.24g/0.09g)

Figure S6. Discharge/charge voltage curves of HPGA-20 and GA anode at 0.1 A/g with a voltage window of 0.1-3.0 V.

Figure S1. Digital Photos and SEM images of the precursors of HPGA-50 after the hydrothermal process (a, b), after the annealing process (c, d), and acid washing process (e, f).


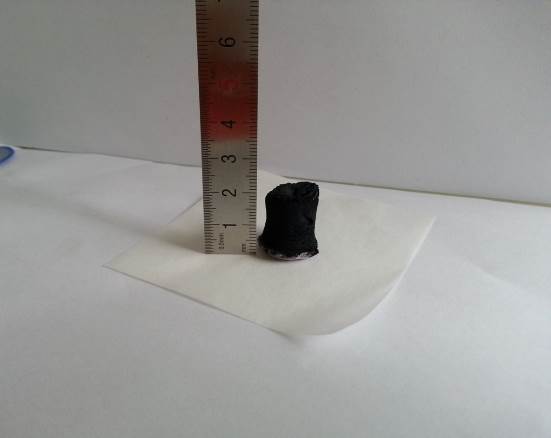

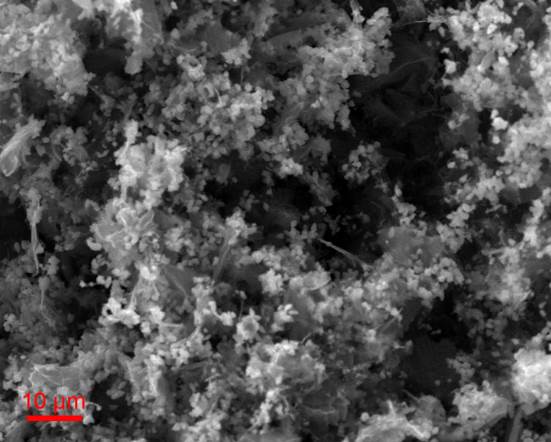


**(a)**

**(b)**


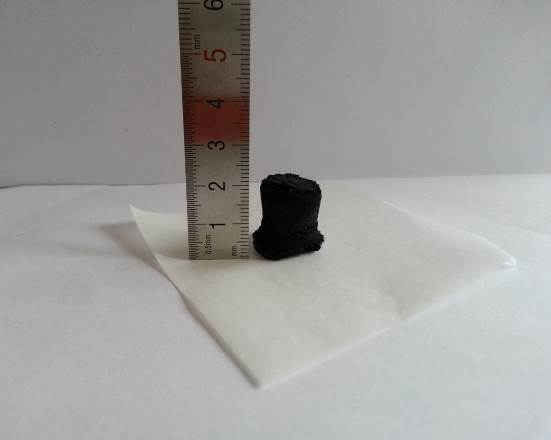

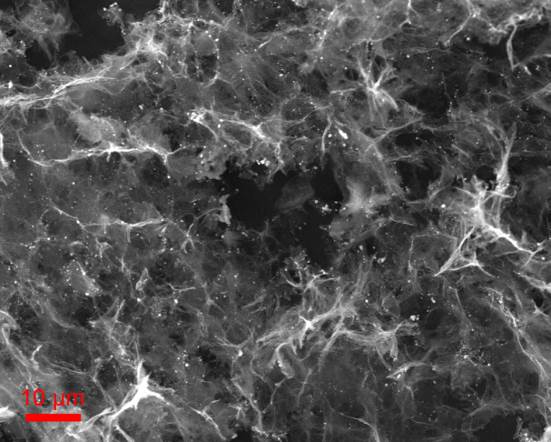


**(c)**

**(d)**


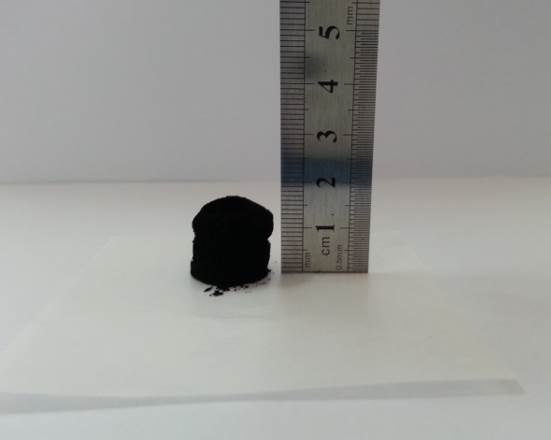

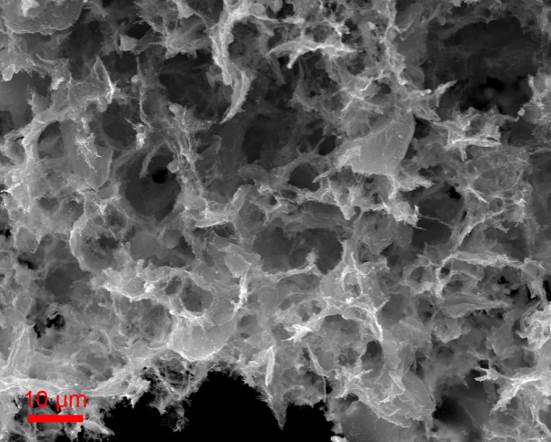


**(e)**

**(f)**

Figure S2. Digital Photos and SEM images of GA without meso-pore graphene sheets after the hydrothermal assembly process of graphene oxide.


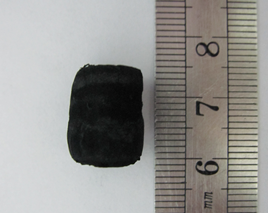

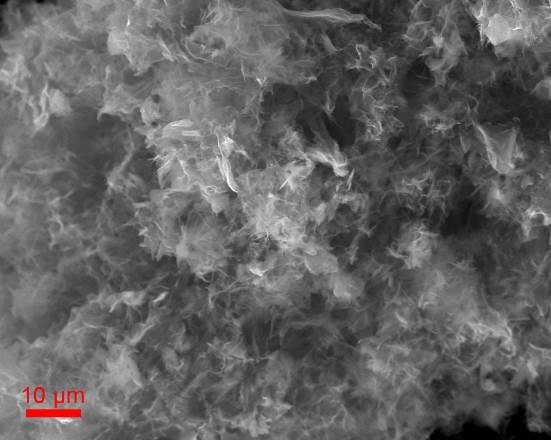


GA


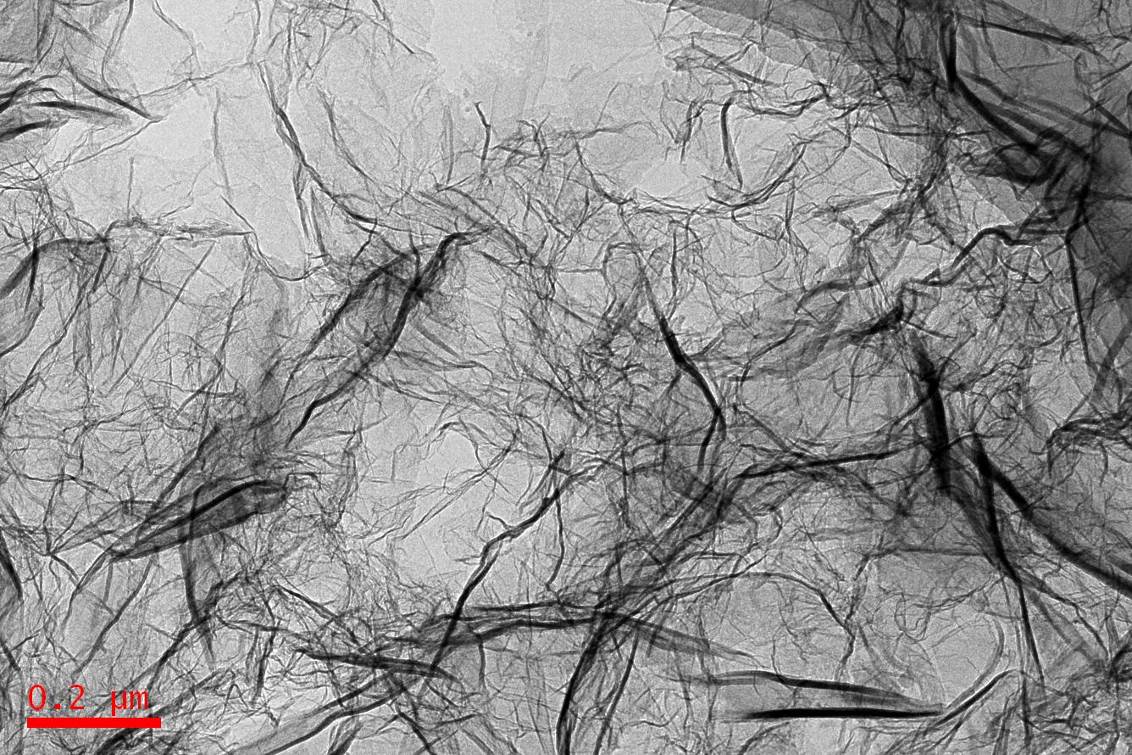


Figure S3. TEM images and statistical pore size results of HPGA-50 (a, b) and HPGA-20 (c, d).


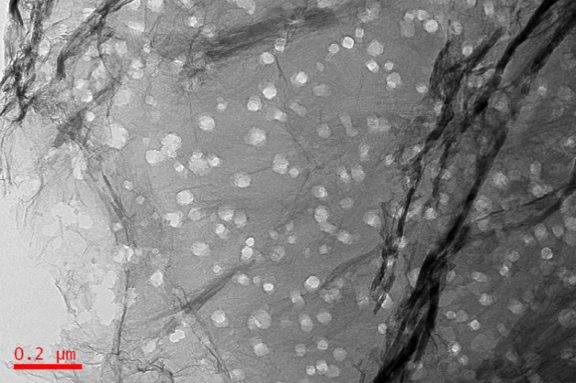

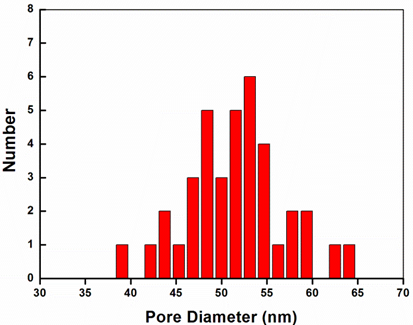


**(a)**

**(b)**


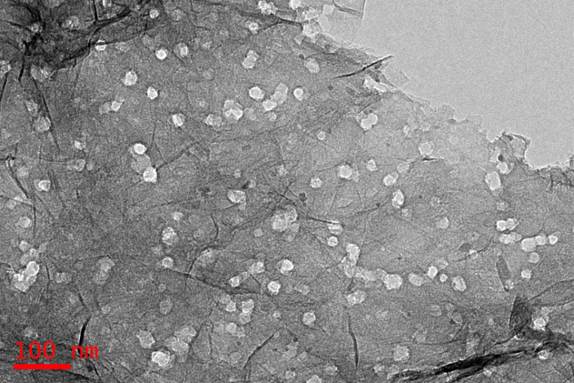

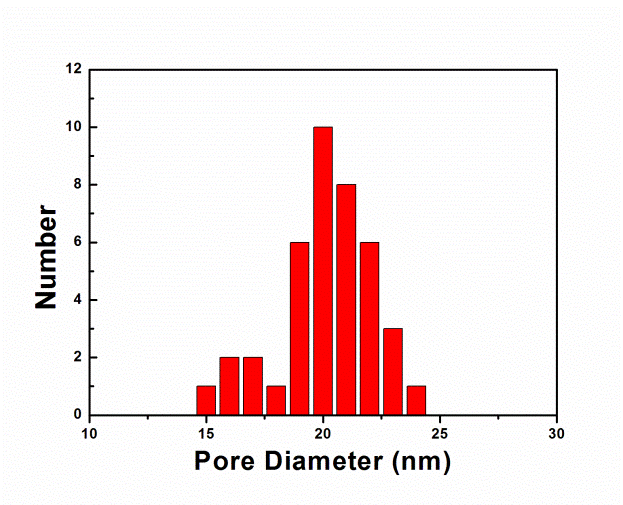


**(c)**

**(d)**

Figure S4. XRD patterns of the as-prepared sample in different stages.


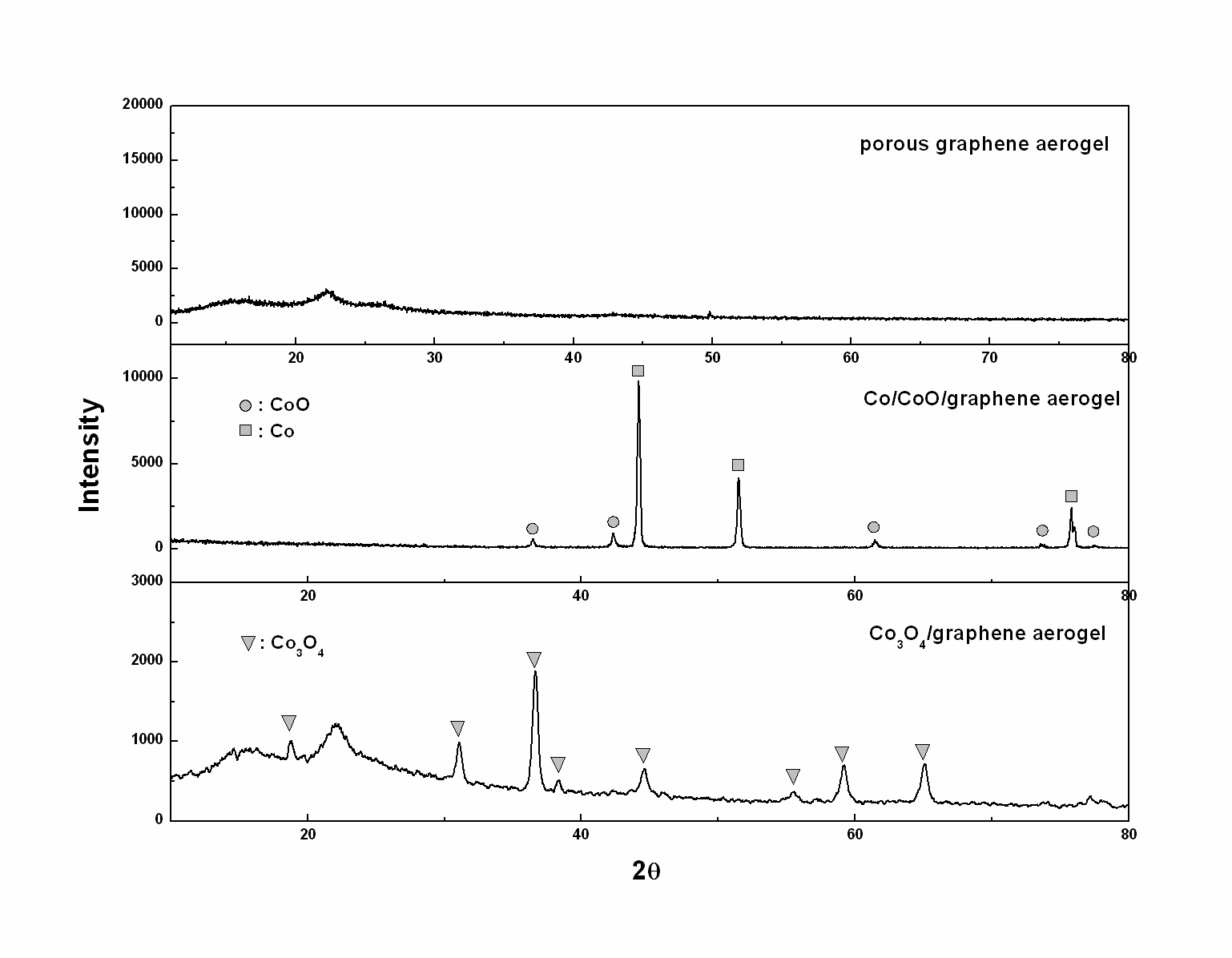


Figure S5. TEM images of HPGA samples prepared by 50 nm Co_3_O_4_ nanoparticles but different Co_3_O_4_/GO ratio (weight ratio: (a) 0.06g/0.09g, (b) 0.12g/0.09g, (c) 0.24g/0.09g). The density of meso-pores on the graphene nanosheets increased with the amount of Co_3_O_4_ nanoparticles used in the experiment.


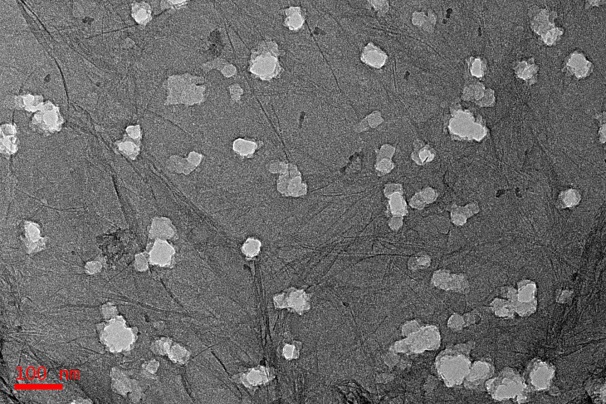

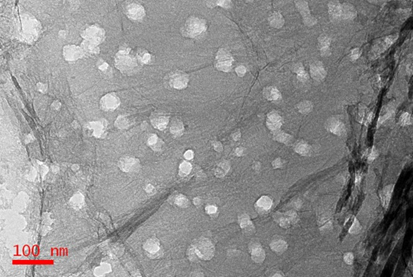

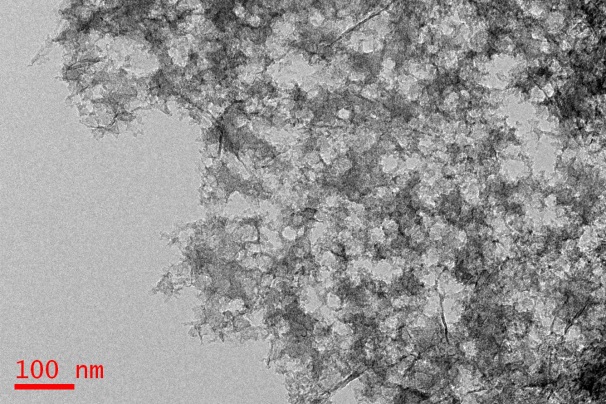


**(a)**

**(b)**

**(c)**

Figure S6. Discharge/charge voltage curves of HPGA-20 and GA anode at 0.1 A/g with a voltage window of 0.1-3.0 V.


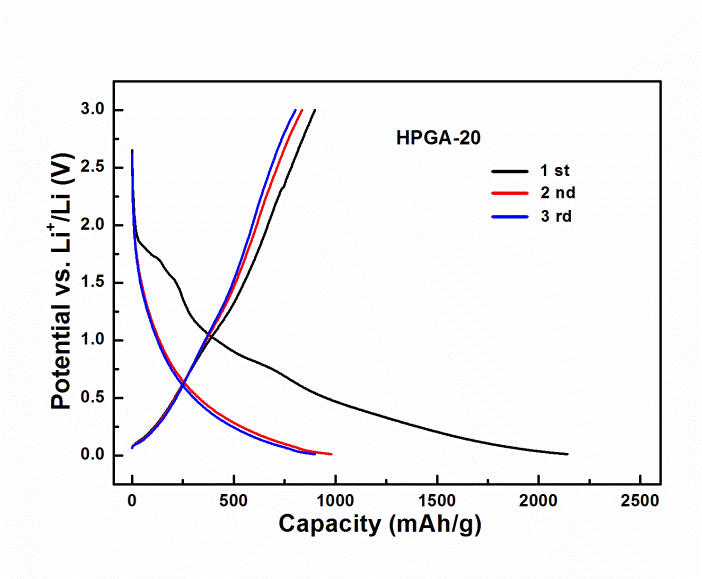

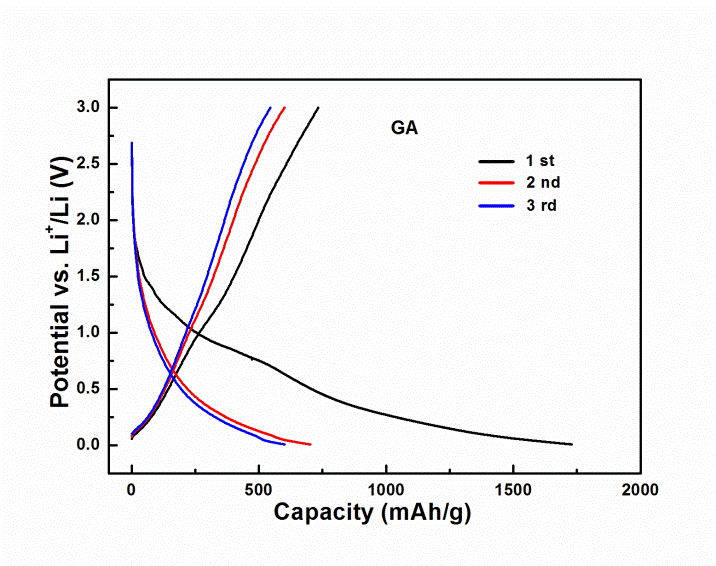


**(a)**

**(b)**
